# Opportunistic Visitors: Long-Term Behavioural Response of Bull Sharks to Food Provisioning in Fiji

**DOI:** 10.1371/journal.pone.0058522

**Published:** 2013-03-13

**Authors:** Juerg M. Brunnschweiler, Adam Barnett

**Affiliations:** 1 Independent Researcher, Zurich, Switzerland; 2 School of Life and Environmental Sciences, Deakin University, Burwood, Victoria, Australia; 3 Fisheries, Aquaculture and Coasts Centre, Institute for Marine and Antarctic Studies, Hobart, Tasmania, Australia; Institute of Marine Research, Norway

## Abstract

Shark-based tourism that uses bait to reliably attract certain species to specific sites so that divers can view them is a growing industry globally, but remains a controversial issue. We evaluate multi-year (2004–2011) underwater visual (n = 48 individuals) and acoustic tracking data (n = 82 transmitters; array of up to 16 receivers) of bull sharks *Carcharhinus leucas* from a long-term shark feeding site at the Shark Reef Marine Reserve and reefs along the Beqa Channel on the southern coast of Viti Levu, Fiji. Individual *C. leucas* showed varying degrees of site fidelity. Determined from acoustic tagging, the majority of *C. leucas* had site fidelity indexes >0.5 for the marine reserve (including the feeding site) and neighbouring reefs. However, during the time of the day (09:00–12:00) when feeding takes place, sharks mainly had site fidelity indexes <0.5 for the feeding site, regardless of feeding or non-feeding days. Site fidelity indexes determined by direct diver observation of sharks at the feeding site were lower compared to such values determined by acoustic tagging. The overall pattern for *C. leucas* is that, if present in the area, they are attracted to the feeding site regardless of whether feeding or non-feeding days, but they remain for longer periods of time (consecutive hours) on feeding days. The overall diel patterns in movement are for *C. leucas* to use the area around the feeding site in the morning before spreading out over Shark Reef throughout the day and dispersing over the entire array at night. Both focal observation and acoustic monitoring show that *C. leucas* intermittently leave the area for a few consecutive days throughout the year, and for longer time periods (weeks to months) at the end of the calendar year before returning to the feeding site.

## Introduction

Some species popular for wildlife-tourism are elusive and can be difficult to find, hence providing food is often used by tourists and tour operators to attract target animals to increase the likelihood of viewing opportunities [1]. The practice of humans feeding wildlife as a tourism attraction is a most controversial issue which is debated highly ideologically and little consensus exists on how the feeding of wildlife for tourism purposes should be managed [2]. In the marine realm, the most prominent examples of this highly controversial issue emerge from shark-feeding dives. Shark-based tourism operations often use bait or chum to reliably attract certain species to specific sites so that divers can view them [3]. This form of shark-based tourism has been growing around the globe in recent years, both in terms of the number of operations in existence and the financial revenue they generate [4], [5]. However, the controversial use of bait and chum to attract sharks for viewing by divers and also the subsequent feeding of bait to sharks has led to concerns that this may be negatively impacting targeted sharks in a number of ways.

To date, research on elasmobranchs (sharks and rays) has shown that baiting and supplemental food provisioning can affect the behaviour, physiology and health of the animals [6]–[12]. Less is known about what effects food provisioning has on the long-term behavioural responses, such as residency patterns and site fidelity of species and individual sharks to specific food provisioning sites, or the associated long-term movement patterns. Using acoustic monitoring, Clarke et al. [13] showed that frequent baiting of silky sharks *Carcharhinus falciformis* leads to increased time spent in the vicinity of the feeding site, but residency patterns varied considerably, from individuals being present almost year-round to others visiting only intermittently. Hammerschlag et al. [14] rejected behaviourally mediated effects of provisioning ecotourism at large spatial and temporal scales in tiger sharks *Galeocerdo cuvier* satellite tagged in the Bahamas. Using focal observation, Clua et al. [15] found a general trend of increasing residency of sicklefin lemon sharks *Negaprion acutidens* at a shark feeding site at Moorea Island. Lastly, Maljković & Côté [16], using both focal observation and acoustic telemetry, found sighting frequencies of individual Caribbean reef sharks *Carcharhinus perezi* to vary widely at a long-term shark provisioning site in the Bahamas, and no effect of time of day on residency at the feeding site.

Long-term shark tourism sites offer platforms to collect baseline data, test specific hypotheses and more generally address a number of questions such as how often, when and for how long individual sharks visit food provisioning sites. At the Shark Reef Marine Reserve in Fiji, where up to eight different species of sharks have been hand-fed since 1999 [4], [17], the bull shark *Carcharhinus leucas* is the numerically dominant species that can be encountered year-round, but with decreasing numbers over the course of a calendar year [18]. The species' seasonal departure from the feeding site is suggested to be related to reproductive activity, but it remains unknown if individuals, many of which can be reliably identified by divers using visible marks and pigmentation [18], leave the area on large-scale or simply stay away from the feeding site or out of sight at certain times of the year. Such information is crucial to obtain in order to eventually identify mating and nursery areas of *C. leucas* in Fiji and to design and implement effective conservation measures. 

In the present study, we evaluate multi-year underwater visual and acoustic tracking data of *C. leucas* from the Shark Reef Marine Reserve. Our specific aims were to 1) determine if *C. leucas* individuals are permanent residents at the Shark Reef Marine Reserve and if not where they are when not present at Shark Reef, 2) define the degree of site fidelity this species shows to the feeding site, and 3) determine if *C. leucas* use the Shark Reef Marine Reserve in similar ways on days when food is offered compared to non-feeding days. Our results provide insight into how often, when and for how long individual *C. leucas* visit the feeding site in the Shark Reef Marine Reserve and surrounding areas, and in so doing provide information on the long-term behavioural response of a charismatic coastal shark species to food provisioning.

## Materials and Methods

### Ethics Statement

Field work was carried out in the Shark Reef Marine Reserve. Research methods included direct observation, stomach and external tagging of free-swimming *C. leucas* (see [18]–[20] for data collected with these methods in the Shark Reef Marine Reserve), and were approved and conducted under a verbal permit provided by the Fijian Ministry of Fisheries and Forestry and with the knowledge and permission of the traditional owners of Shark Reef.

### Study Area, Dive Protocol, Focal Observation and Shark Behaviour

**Figure 1:**
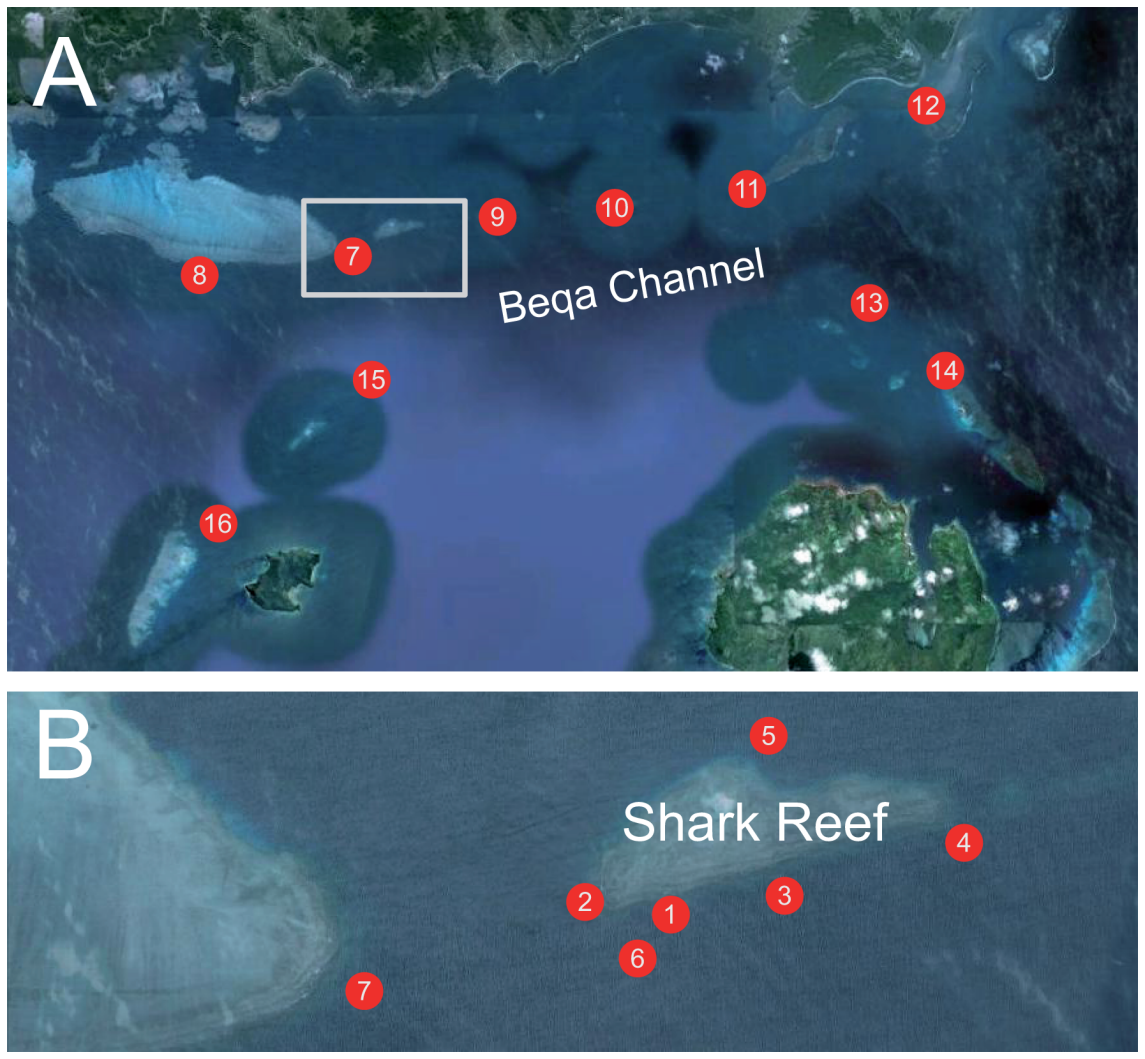
Receiver array on the southern coast of Viti Levu, Fiji. (A) Off Shark Reef (inset) stations (7–16), and (B) locations of Shark Reef stations (1–6).

Shark Reef is located on the southern coast of Viti Levu, Fiji [4]. This small reef patch is part of a fringing reef that is separated from the shallow waters of Beqa Lagoon by the deep (∼250 m) Beqa Channel (Fig. 1). Focal observations of *C. leucas* were made between 2003 and 2011 on commercial shark watching dives in the Shark Reef Marine Reserve, following the dive, feeding and data collection protocol described in Brunnschweiler & Baensch [18]. Briefly, sharks have been hand-fed on three to five days per week between 09:00 and 12:00 at different sites within the marine reserve. The feeding sites are about 20–30 m from one another at different depths on the ocean facing side of the reef that slopes down from the reef crest just below the water surface into the Beqa Channel. The dive procedure starts with a first dive to 30 m where sharks are attracted and fed with fish scraps and/or whole fish heads. At this feeding site, only *C. leucas* and tawny nurse sharks *Nebrius ferrugineus* turn up regularly. After 17 minutes, the divers ascend up the reef slope to shallower waters where the feeder hand-feeds grey reef *Carcharhinus amblyrhynchos*, whitetip reef *Triaenodon obesus* and blacktip reef sharks *Carcharhinus melanopterus*. After a one hour surface interval, a second dive is conducted at 16 m where the feeder hand-feeds *C. leucas* and, if present, other large species with whole fish heads.

Focal observations in the Shark Reef Marine Reserve have been made since 2003 by a few trained observers including one of us (JMB). These show that *C. leucas* only gradually show up on the first dive of the day at 30 m, presumably ascending from the deeper waters of the Beqa Channel, and in lower numbers compared to the second dive of the day at 16 m (Fig. S1). At both feeding sites, however, *C. leucas* disappear out of sight immediately after feeding has stopped and the divers leave the area. During non-provisioning dives at the Shark Reef Marine Reserve, *C. leucas* have been encountered occasionally in low numbers and only for short time periods at the beginning of the dive.

To date, >100 *C. leucas* individuals have been catalogued in the Shark Reef Marine Reserve database using visible marks and pigmentation [18]. For 48 of these that have been encountered since 2009 or before (17 since 2004, 7 since 2007, 4 since 2008, 20 since 2009; Table S1), we quantified the degree of residency to the feeding site by dividing the number of days an individual was observed by the number of days such data were collected at the Shark Reef Marine Reserve (site fidelity index – SFI_v_). Values of site fidelity range from 0 to 1, with values close to 0 indicating low site fidelity and values close to 1 indicating high site fidelity. Depending on the uniqueness and/or obviousness of marks and pigmentation, identification of individuals can be challenging and correct identification levels may differ between observers. In order to quantify this challenge, we asked both a trainee and the senior observer, who established the database in 2003 and has named all the catalogued sharks, to rate each of the 48 individual as ‘unequivocal’, ‘easy’ or ‘challenging’ to identify. In those cases where the two observers disagreed on the level of difficulty to identify an individual (29.2%) or where the trainee had not yet encountered the individual (18.8%), we used the more challenging rating or the senior observer's judgment, respectively, for further analyses.

### Acoustic Monitoring

Between 2006 and 2010, adult *C. leucas* visiting the feeding sites in the Shark Reef Marine Reserve [18] were tagged with a total of 103 acoustic-coded Vemco R-coded, 69 kHz acoustic transmitters (min/max off times between 15–45 s and 110–250 s) manufactured by Amirix Systems Inc., Nova Scotia, Canada (Table 1). Sharks were tagged predominantly at the beginning of a calendar year (January–March), when *C. leucas* numbers are highest in the Shark Reef Marine Reserve, or in late August/early September (Fig. S2), the time of the year numbers start to decrease [18].

**Table 1:** *Carcharhinus leucas* (n = 82) acoustically tagged at the Shark Reef Marine Reserve between 2006 and 2010.

Transmitter ID	Transmitter type	Name	Sex	Method	Tagging date	Number of days monitored	Number of stations	Mean number of detections per day
1	V16 (570)	Whitenose	M	1	17 March 2006	7	**4** (1,2,9,10)	10.9
2	V16 (570)	UKN	F	1	17 March 2006	9	**4** (1,2,9,10)	26.2
3	V16 (570)	UKN	F	1	18 March 2006	7	**4** (1,2,9,10)	44.3
4	V16 (570)	UKN	F	1	18 March 2006	3	**4** (1,2,9,10)	40.3
5	V16 (570)	Stumpy	F	1	20 March 2006	11	**4** (1,2,9,10)	2.9
7	V16 (570)	Crook	F	1	19 May 2006	2	**3** (1,9,10)	108
8	V16 (641)	Hook	F	1	23 June 2006	4	**3** (1,9,10)	51.5
9	V16 (570)	UKN	F	1	2 January 2007	5	**5** (1,2,9,10,15)	38.4
10	V16 (641)	UKN	M	1	3 January 2007	19	**5** (1,2,9,10,15)	52.4
11	V16 (641)	UKN	F	1	3 January 2007	6	**5** (1,2,9,10,15)	268.7
12	V16 (641)	Monica	F	1	4 January 2007	11	**5** (1,2,9,10,15)	83.9
13	V16 (641)	Crook	F	1	6 January 2007	16	**5** (1,2,9,10,15)	37.7
14	V16 (641)	Flop	F	1	6 January 2007	1	**5** (1,2,9,10,15)	17
15	V16 (641)	UKN	M	1	8 January 2007	13	**5** (1,2,9,10,15)	67.4
16	V16 (641)	Bum	F	1	9 January 2007	8	**5** (1,2,9,10,15)	43.5
17	V16 (641)	UKN	F	1	10 January 2007	1	**5** (1,2,9,10,15)	76
18	V16T (770)	Valerie	F	1	28 January 2008	2	**8** (1–5, 8,10,15)	3
19	V16T (770)	Grin	F	1	28 January 2008	9	**8** (1–5, 8,10,15)	35.1
21	V16T (770)	Whitenose	M	1	1 February 2008	2	**8** (1–5, 8,10,15)	40.5
22	V16 (770)	Monica	F	1	5 February 2008	7	**10** (1–5,8,10,11,13,15)	101.7
23	V16 (770)	Bum	F	1	5 February 2008	5	**10** (1–5,8,10,11,13,15)	15.6
25	V16 (770)	Hotlips	F	1	8 February 2008	4	**10** (1–5,8,10,11,13,15)	68.8
26	V16 (770)	Hook	F	1	13 February 2008	2	**10** (1–5,8,10,11,13,15)	76
27	V16 (770)	Chopper	M	1	15 February 2008	7	**10** (1–5,8,10,11,13,15)	18.7
28	V16 (770)	UKN	M	2	23 February 2008	11	**10** (1–5,8,10,11,13,15)	3.2
29	V16 (770)	Bumphead	F	2	23 February 2008	63	**10** (1–5,8,10,11,13,15)	63
30^a^	V16 (770)	Bumphead	F	1	1 March 2008	9	**10** (1–5,8,10,11,13,15)	17.8
32	V16 (770)	Rip	F	2	3 March 2008	462	**16** (1–16)	51
33	V16 (770)	UKN	F	2	3 March 2008	34	**10** (1–5,8,10,11,13,15)	38.4
34	V16 (770)	Monica	F	1	5 March 2008	2	**10** (1–5,8,10,11,13,15)	7.5
35	V16 (770)	Chopper	M	2	5 March 2008	4	**10** (1–5,8,10,11,13,15)	4.8
37	V16 (770)	UKN	F	2	19 Mach 2008	17	**10** (1–5,8,10,11,13,15)	23.2
38	V16 (770)	UKN	F	2	19 March 2008	20	**10** (1–5,8,10,11,13,15)	5.6
39	V16 (770)	Flop	F	1	21 March 2008	1	**9** (1–5,8,11,13,15)	48
40	V16 (770)	Bum	F	1	21 March 2008	1	**9** (1–5,8,11,13,15)	15
42	V16 (770)	Hotlips	F	1	26 March 2008	2	**9** (1–5,8,11,13,15)	22.5
43	V16 (770)	UKN	F	2	26 March 2008	1	**9** (1–5,8,11,13,15)	4
44	V16 (770)	Second	F	1	29 March 2008	1	**9** (1–5,8,11,13,15)	30
45	V16 (770)	UKN	F	2	29 March 2008	13	**9** (1–5,8,11,13,15)	28.8
46	V9T (73)	Crook	F	1	28 June 2008	6	**10** (1–5,8,10,11,13,15)	21.5
47	V9T (73)	Hotlips	F	1	2 July 2008	5	**10** (1–5,8,10,11,13,15)	14.4
50	V9T (73)	Monica	F	1	25 July 2008	13	**10** (1–5,8,10,11,13,15)	0.8
55	V16 (1157)	UKN	F	2	25 August 2008	68	**14** (1–5,7–15)	49.4
56	V16 (1157)	UKN	M	2	25 August 2008	32	**14** (1–5,7–15)	24.8
57	V16 (1157)	UKN	F	2	26 August 2008	5	**13** (1–5,7–13,15)	7.8
59	V16 (1157)	UKN	F	2	28 August 2008	145	**13** (2–5,7–15)	11.2
60	V16 (1157)	UKN	F	2	28 August 2008	24	**13** (2–5,7–15)	8.2
61	V16 (1157)	UKN	F	2	30 August 2008	12	**13** (2–5,7–15)	22.3
62	V16 (1157)	UKN	M	2	30 August 2008	36	**13** (2–5,7–15)	13.7
63	V16 (1157)	UKN	F	2	2 September 2008	1	**13** (2–5,7–15)	76
64	V16 (1157)	Granma	F	2	2 September 2008	25	**13** (2–5,7–15)	17
66	V16 (1159)	UKN	F	2	5 February 2009	12	**15** (1–15)	52.8
67	V16 (1159)	UKN	M	2	5 February 2009	109	**16** (1–16)	48.7
68	V16 (1159)	UKN	F	2	5 February 2009	8	**15** (1–15)	32.1
69	V16T (448)	Chopper	M	1	7 February 2009	28	**15** (1–15)	39
70^b^	V16T (448)	Crook	F	1	7 February 2009	10	**15** (1–15)	51.7
71	V16T (448)	Whitenose	M	1	7 February 2009	6	**15** (1–15)	48
72	V16T (448)	Blunt	F	1	7 February 2009	8	**15** (1–15)	141.6
73	V16 (1159)	UKN	F	2	7 February 2009	239	**16** (1–16)	31.4
74	V16 (1159)	UKN	M	2	7 February 2009	11	**15** (1–15)	87
75	V16 (1159)	Hook	F	2	7 February 2009	355	**16** (1–16)	46
76	V16T (448)	Scar	F	1	9 February 2009	7	**15** (1–15)	193.9
77	V16T (448)	Grin	F	1	9 February 2009	5	**15** (1–15)	77.2
78	V16 (1159)	UKN	M	2	9 February 2009	105	**16** (1–16)	27.4
79	V16 (1159)	UKN	F	2	9 February 2009	119	**16** (1–16)	60.2
80	V16 (1159)	Crook	F	2	10 February 2009	486	**16** (1–16)	17.9
81	V16T (448)	UKN	F	1	11 February 2009	8	**15** (1–15)	106.5
82	V16T (448)	Bum	F	1	11 February 2009	1	**15** (1–15)	65
83	V16 (1159)	Whitenose	M	2	11 February 2009	184	**16** (1–16)	24.2
84	V16T (448)	Maite	F	1	13 February 2009	1	**15** (1–15)	54
85	V16 (1159)	UKN	F	2	17 August 2009	26	**16** (1–16)	44.9
86	V16P (1122)	UKN	M	2	17 August 2009	51	**16** (1–16)	77.8
89	V16P (1122)	Wave	F	2	18 August 2009	27	**16** (1–16)	23.8
91	V16P (1122)	UKN	F	2	20 August 2009	19	**16** (1–16)	43.6
93	V16P (1122)	UKN	F	2	25 August 2009	57	**16** (1–16)	25.5
95	V13 (1585)	Chopper	M	1	22 February 2010	6	**16** (1–16)	6
96	V13 (1585)	Grin	F	1	22 February 2010	5	**16** (1–16)	24
97	V13 (1585)	Sierra	F	1	23 February 2010	1	**16** (1–16)	25
98	V13 (1585)	Marlen	F	1	23 February 2010	4	**16** (1–16)	2.3
99	V13 (1585)	Whitenose	M	1	25 February 2010	11	**16** (1–16)	14.1
100	V13 (1585)	Bum	F	1	25 February 2010	2	**16** (1–16)	2
103^c^	V13 (1585)	Crook	F	1	4 March 2010	3	**16** (1–16)	303

Sharks were tagged with V9, V13 or V16 acoustic transmitters (9 transmitters attached externally with a pressure sensor (V16P), 22 transmitters with a temperature sensor (VxT) fed to *C. leucas* to monitor stomach temperature). Estimated tag life (days) is given in brackets. UKN  =  unknown, method 1 = tag hand-fed, method 2 = tag externally attached. Bold numbers are maximum number of receivers in the water during the monitoring period with individual station numbers in brackets. Refer to Table S1 in [18] for the description of the natural marks of visually identifiable *C. leucas* individuals (n = 21).

a Double-tagged with transmitter ID 29; excluded from analysis except for monitoring period

b Double-tagged with transmitter ID 80 10–17 February 2009; excluded from analysis except for monitoring period

c Double-tagged with transmitter ID 80; excluded from analysis except for monitoring period

Shark Reef is a protected area in which the catching of marine life is not permitted, therefore we chose to either hand-feed transmitters to sharks (stomach tags; n = 62) at the 16 m feeding site [19] or have tags externally attached (external tags; n = 41) by a scuba diver using a spear gun [20]. Of all *C. leucas* individuals tagged, 21 could be identified using marks and pigmentation [18]; 10 of these were double-tagged or tagged multiple times (Table 1). One female (ID 32) was double-tagged with a pop-up satellite archival tag (PSAT) for 9 days in 2009 (7–16 February; F14 in [20]).

The coarse-scale movements of tagged *C. leucas* and their site fidelity to Shark Reef, specifically to the feeding sites within the Shark Reef Marine Reserve, were quantified using an array of up to 16 Vemco VR2/VR2W omnidirectional acoustic receivers (Amirix Systems Inc., Nova Scotia, Canada) deployed at various reef locations (Fig. 1). Receivers were either attached to mooring lines or a rope of 1.5–3 m length with a floating buoy and attached to a cement block. Shark Reef was equipped with at maximum six acoustic receivers (Stations 1–6; Fig. 1, Table 2). Being attached to the dive boat mooring line at 18 m depth in front and above the 16 m and 30 m feeding sites, respectively, Station 1 was considered the major (only) baiting station. Most receivers were placed on the reef slope facing Beqa Channel or channels between reef patches (e.g. Stations 2 and 7) at depths of 15–40 m, with the exception of station 5 which was placed on the inside of Shark Reef and Station 12 placed at the mouth of the Navua River (Fig. 1). Four receivers (13–16) were placed at or near reef patches on the other side of Beqa Channel (Fig. 1). Acoustic receiver locations were generally chosen to detect shark movements along the fringing reefs and across the Beqa Channel (Stations 13–16), and also to provide monitoring coverage of deeper areas (e.g. Station 6). Maximum depths at which *C. leucas* were detected at particular receivers ranged from 22.7 m (Station 14) to 146.5 m (Station 6; Table 2). Receiver detection range tests were carried out in the array in February 2008 and May 2010 and yielded estimates of generally <60 m, values typical for coral reef habitats [21], [22]. Stations 1, 2, 3 and 6 had partially overlapping detection ranges.

**Table 2:** Coverage periods of acoustic receivers used in the study.

Station number	Start coverage	End coverage	Periods not covered (days)	Maximum depth (m)	Mean depth (m)
Shark Reef					
1	17 March 2006	End of study	27 August 2008 – 4 February 2009 (161)	104.6	36.4 (10.3)
			16 May – 17 June 2009 (32)		
2	17 March 2006	End of study	21 March 2006 – 5 January 2007 (290)	123.7	41 (18.5)
3	15 January 2008	12 February 2010		126.4	38.6 (12.3)
4	22 January 2008	End of study		NA	NA
5	22 January 2008	26 February 2010		NA	NA
6	12 February 2009	End of study		146.5	39.3 (12.6)
Off Shark Reef					
7	27 August 2008	End of study	27 November 2009 – 15 January 2010 (49)	52.8	21.8 (8.9)
8	22 January 2008	End of study	17 June – 21 August 2009 (65)	65.5	46 (14.8)
9	18 March 2006	End of study	28 January – 27 August 2008 (212)	NA	NA
			25 August – 15 September 2009 (21)		
10	18 March 2006	End of study	20 March – 21 May 2008 (62)	78.2	30.1 (17.6)
11	9 February 2008	End of study		105.5	34.3 (18.1)
12	29 August 2008	End of study		28.2	21.7 (4.2)
13	9 February 2008	End of study		NA	NA
14	2 September 2008	End of study		22.7	21.7 (4.2)
15	18 September 2006	End of study		40	24.2 (9)
16	17 April 2009	End of study		NA	NA

Maximum and mean (±SD) depths recorded at particular receivers were determined from *C. leucas* equipped with V16P transmitters (Table 1). NA  =  not availabl

### Acoustic Monitoring Analysis

Presence of *C. leucas* was examined daily, with tagged individuals considered present in the array (all stations), at Shark Reef (SR; Stations 1–6) or off SR (Stations 7–16) if two or more detections were heard on any of the respective receivers on a given day. Transmitters that were either not heard from or were only logged at the day of tagging were not included in the analysis (n = 21). For *C. leucas* being considered present at a particular receiver (e.g. at Station 1) and during a particular time period (e.g. present at Station 1 between 09:00 and 12:00), at least two detections had to be logged at such receiver on a given day or during the time period, respectively. The mean number of detections per day was calculated by dividing the total number of detections by the monitoring period of the individual.

For *C. leucas* monitored for >10 days (n = 36; Table 3), we calculated site fidelity indexes (SFI_a_) for Array (all stations), SR (Stations 1–6), 1 (Station 1) and 1_9-12_ (Station 1 between 09:00 and 12:00) by dividing the number of days an individual was detected in the Array, at SR, Station 1 or Station 1 between 09:00 and 12:00 by the monitoring period of the individual. In order to evaluate whether or not *C. leucas* came to the feeding site more often on days when food was offered compared to non-feeding days, we divided the number of days an individual was detected at Station 1 between 09:00 and 12:00 by the number of feeding days (1_9-12feeding_) and non-feeding days (1_9-12non-feeding_), respectively.

**Table 3:** SFI_a_ values determined from transmitters attached to *C. leucas* for >10 days (n = 36).

Transmitter ID	Name	Number of days monitored	SFI_a_					
			Array	SR	1	1_9-12_	1_9-12feeding_	1_9-12non-feeding_
5	Stumpy	11	0.92	0.67	0.67	0.25	0.29	0.2
12	Monica	11	1	1	1	0.92	0.9	1
28	UKN	11	0.25	0.25	0.25	0.17	0.29	0
74	UKN	11	1	0.92	0.92	0.75	0.75	0.75
99	Whitenose	11	1	1	0.67	0.5	0.75	0
61	UKN	12	0.69	0.62	NA	NA	NA	NA
66	UKN	12	1	0.92	0.69	0.54	0.44	0.75
15	UKN	13	0.79	0.79	0.79	0.79	0.8	0.75
45	UKN	13	0.79	0.79	0.71	0.5	0.44	0.6
50	Monica	13	0.14	0.14	0.07	0.07	0.11	0
13	Crook	16	0.94	0.82	0.65	0.53	0.54	0.5
37	UKN	17	0.72	0.72	0.67	0.61	0.57	0.75
10	UKN	19	0.8	0.75	0.7	0.65	0.69	0.5
91	UKN	19	0.85	0.7	0.6	0.25	0.33	0.13
38	UKN	20	0.24	0.19	0.19	0.19	0.19	0.2
60	UKN	24	0.72	0.24	NA	NA	NA	NA
64	Granma	25	0.85	0.69	NA	NA	NA	NA
85	UKN	26	0.96	0.78	0.67	0.41	0.43	0.36
89	Wave	27	0.54	0.54	0.43	0.29	0.38	0.17
69	Chopper	28	0.66	0.59	0.55	0.52	0.68	0.2
56	UKN	32	0.76	0.7	NA	NA	NA	NA
33	UKN	34	0.74	0.66	0.57	0.54	0.62	0.43
62	UKN	36	0.59	0.59	NA	NA	NA	NA
86	UKN	51	0.69	0.63	0.62	0.58	0.57	0.58
93	UKN	57	0.64	0.52	0.43	0.31	0.39	0.22
29	Bumphead	63	0.77	0.73	0.69	0.61	0.69	0.52
55	UKN	68	0.84	0.78	NA	NA	NA	NA
78	UKN	105	0.65	0.61	0.54	0.52	0.67	0.28
67	UKN	109	0.71	0.65	0.57	0.5	0.55	0.42
79	UKN	119	0.51	0.46	0.34	0.31	0.33	0.28
59	UKN	145	0.45	0.3	NA	NA	NA	NA
83	Whitenose	184	0.63	0.58	0.47	0.4	0.42	0.37
73	UKN	239	0.49	0.45	0.35	0.33	0.34	0.32
75	Hook	355	0.46	0.36	0.32	0.29	0.34	0.21
32	Rip	462	0.53	0.47	0.56	0.48	0.48	0.48
80	Crook	486	0.4	0.32	0.25	0.21	0.24	0.16
	Mean (±SD)		0.69 (0.11)	0.61 (0.22)	0.55 (0.21)	0.45 (0.20)	0.49 (0.20)	0.38 (0.26)

Array  =  all stations, SR  =  Stations 1–6, 1  =  Station 1, 1_9-12_ =  Station 1 between 09:00 and 12:00, 1_9-12feeding_  =  Station 1 between 09:00 and 12:00 on feeding days, 1_9-12non-feeding_  =  Station 1 between 09:00 and 12:00 on non-feeding days. NA  =  not available, UKN  =  unknown.

A Fast Fourier Transform (FFT) was computed for 12 of the 16 individuals monitored for >30 days (Table 3) to identify any temporal periodicity in shark activity around Station 1 on feeding and non-feeding days. No Fourier analysis was conducted for the other four *C. leucas* (IDs 55, 56, 59, 62) as Station 1was not in the water for the majority of the detection period for these individuals. Input data were the number of detections per hour blocks. A FFT separates time-series data into frequencies and identifies any sinusoid patterns, or periodicity, in the dataset. A power spectrum is then constructed and the dominant frequencies are represented by peaks in the spectrum [23]. Before analysis, data were smoothed with a Hamming window, a weighted moving average transformation used to smooth the periodogram values [24]. Windowing reduces discontinuity between frames, smoothes the data and reduces noise, thus improving the ‘quality’ of the harmonics so that spectral leakage is reduced and it is easier to identify the frequencies that contribute the most for the overall periodicity of the time series. To further investigate temporal use of Station 1 on feeding and non-feeding days, a timeline using all tagged sharks was constructed showing the hours of the day a shark was present at Station 1. A circular statistics Chi-square test (Oriana 3 software) was used to compare if there were differences in arrival times of *C. leucas* at Station1 between feeding and non-feeding days. The number of consecutive hours sharks spent at Station 1 was also compared between feeding and non-feeding days with the non-parametric Mann-Whitney *U*-test. Only data for animals first detected in the morning (during potential feeding period) were considered, i.e. only animals/days for which the first detections were between 09:00 and 12:00 were used, and each day any individual shark was present was considered as a replicate.

Circular statistics were also used to study the diurnal pattern of area use around stations or groups of stations for *C. leucas* monitored for >10 days (n = 36; Table 3). For these analyses, the response variable was the number of individuals detected by a receiver at each of the 24 hours of the day, and replicates were the different days. If a shark was detected by a receiver two or more times in any particular hour, it was considered as having been present during that hour. Chi-square circular tests were used to examine if there were temporal (over the 24 h day) differences in area use of each station or grouped stations. These comparisons were also conducted on feeding and non-feeding days.

For *C. leucas* monitored for >50 days (n = 13; Table 3), we plotted days that each individual was detected at Shark Reef (Stations 1–6) and off Shark Reef (Stations 7–16) on a timeline to determine if individuals are permanent residents at Shark Reef and to compare detections inside and outside the Shark Reef Marine Reserve. The number of times an individual returned to the receiver array or Shark Reef after it was absent for a period of 24 h or more, respectively, was tallied for each *C. leucas*.

## Results

Of a total of 103 acoustic transmitters attached to *C. leucas* individuals, 82 were included in the analysis with monitoring periods of 1–486 days (total number of detections = 114,282; Table 1, Fig. S2). Forty-nine of these were fed to *C. leucas*, resulting in monitored periods of 1–28 days (mean±SD = 6.3±5.3 days). *Carcharhinus leucas* with tags attached externally (n = 33) were monitored for periods of 1–486 days (mean ± SD = 84.3±126.6 days). The mean number of detections per day of acoustically tagged *C. leucas* in the receiver array ranged from 0.8–268.7 (Table 1).

### Area and Station Use

**Figure 2:**
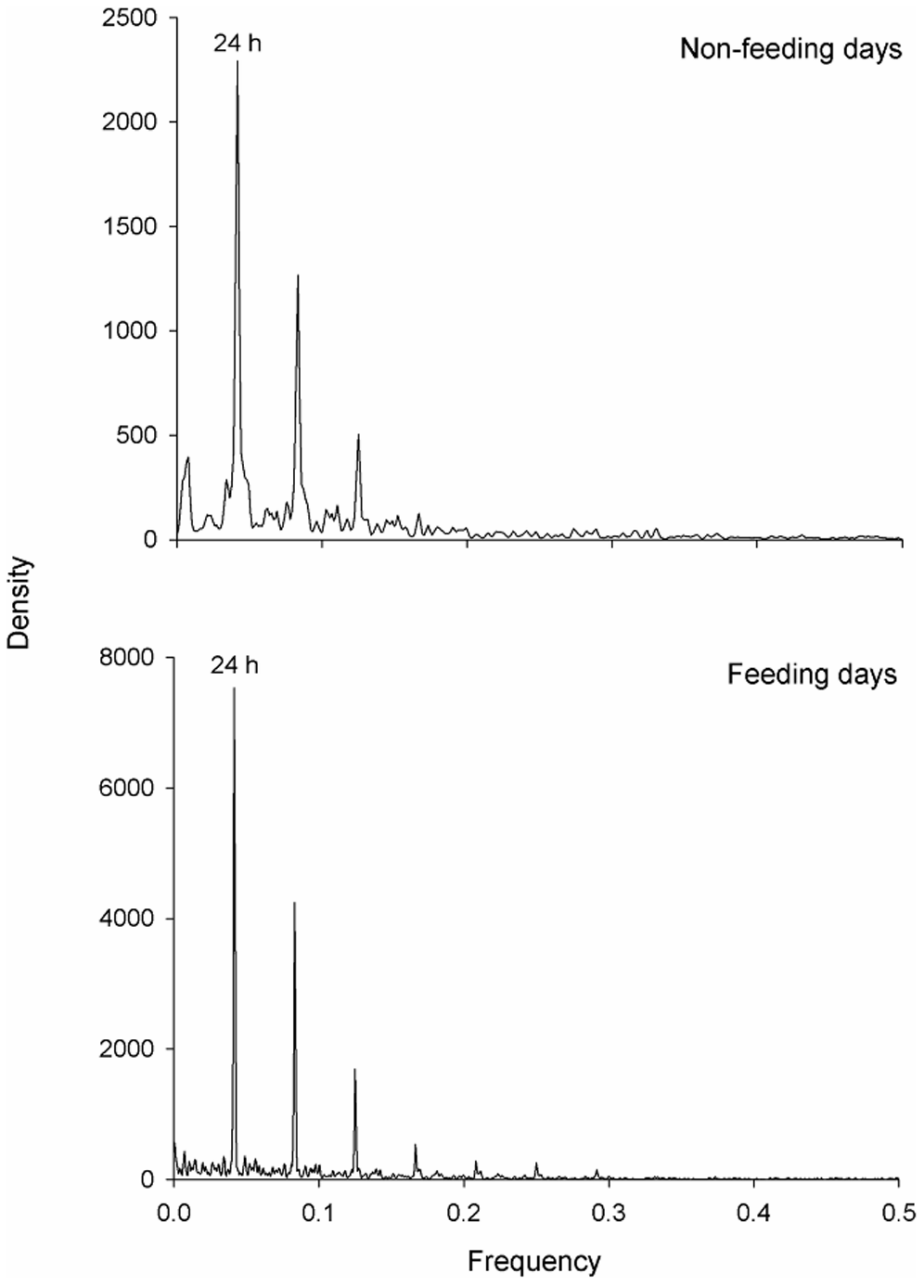
Fast Fourier transform of the time series of number of detections per hour at Station 1 for one representative *C. leucas* individual (ID 75). A 24 h pattern was evident for 11 of the 12 *C. leucas* tested (see text for details). The x-axis shows the frequency, a function of periodicity, and the y-axis is the spectral density, indicating the most important cycle periodicities.

**Figure 3:**
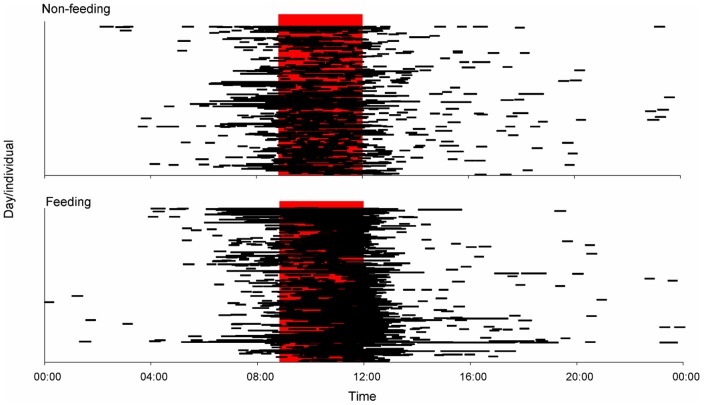
24 h timeline showing the period of time a *C. leucas* was present at Station 1 on non-feeding and feeding days. Each line in the timeline (y-axis) represents an individual's occurrence at Station 1 on a given day. The red shaded area denotes the time of the day when feeding occurred at the Shark Reef Marine Reserve (09:00–12:00).

A circadian (24 h) periodicity in the use of Station 1 was detected for 11 of the 12 *C. leucas* tested both on feeding and non-feeding days (see Fig. 2 for an example). One individual (ID 93) showed no periodicity in the use of Station 1 on either feeding or non-feeding days. Diel timeline graphs (24 h) show that this periodicity is associated with *C. leucas* consistently occurring at Station 1 in the mornings regardless of whether feeding or non-feeding days (Fig. 3) with no significant difference in the arrival time at Station 1 on feeding or non-feeding days (Chi-square test, p = 0.158). However, there was a significant difference in the amount and distribution of hours *C. leucas* spent at Station 1 between feeding and non-feeding days (Chi-square test, p<0.001). The diel timeline graph suggests that this difference is driven by *C. leucas* remaining at Station 1 for longer periods of time on feeding days (Fig. 3). Moreover, after being detected for the first time between 09:00 and 12:00, sharks spent significantly more consecutive hours at Station 1 on feeding days (2.6±1.2 hours) than on non-feeding days (2.0±1.0 hours) (Mann–Whitney *U*-test, p<0.001).

**Figure 4:**
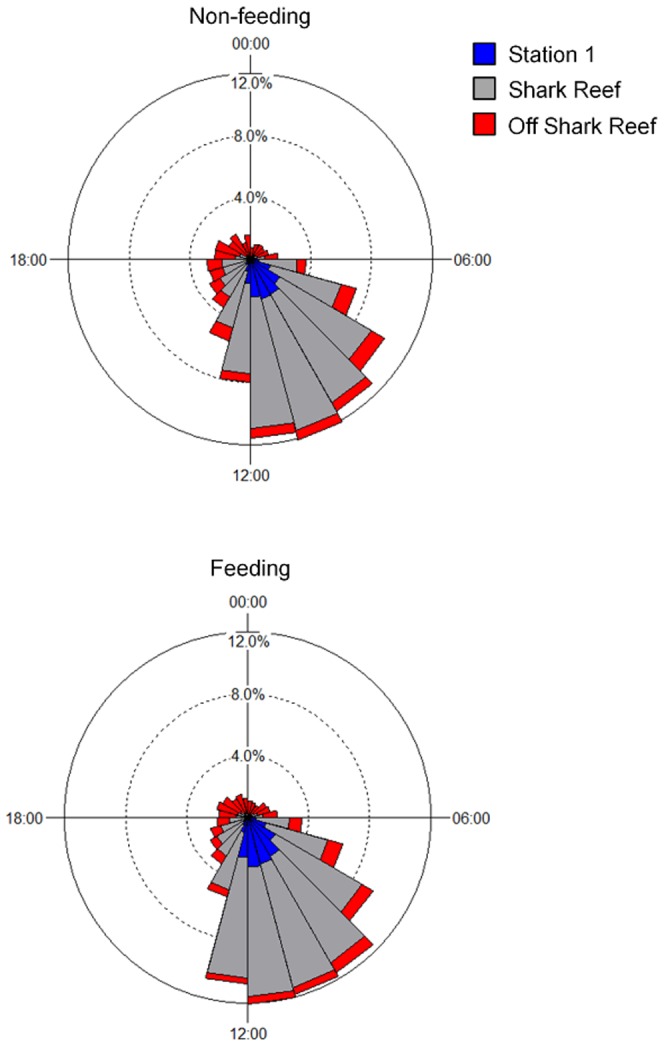
Circular plots showing the percentage of hours *C. leucas* were detected at Station 1, Shark Reef and off Shark Reef over the 24 h diel cycle.

Further comparisons of area use based on stations grouped showed that the use of the remainder of Shark Reef (Stations 2–6 grouped) did not vary between feeding and non-feeding days (Chi-square test, p = 1.000), nor did the use of areas outside of Shark Reef (Stations 7–16 grouped) (Chi-square test, p = 1.000) (Fig. 4). The overall pattern is for *C. leucas* to use the area around Station 1 in the morning before spreading out over Shark Reef throughout the day and dispersing over the entire array at night (Figs. 4 and S3).

**Figure 5:**
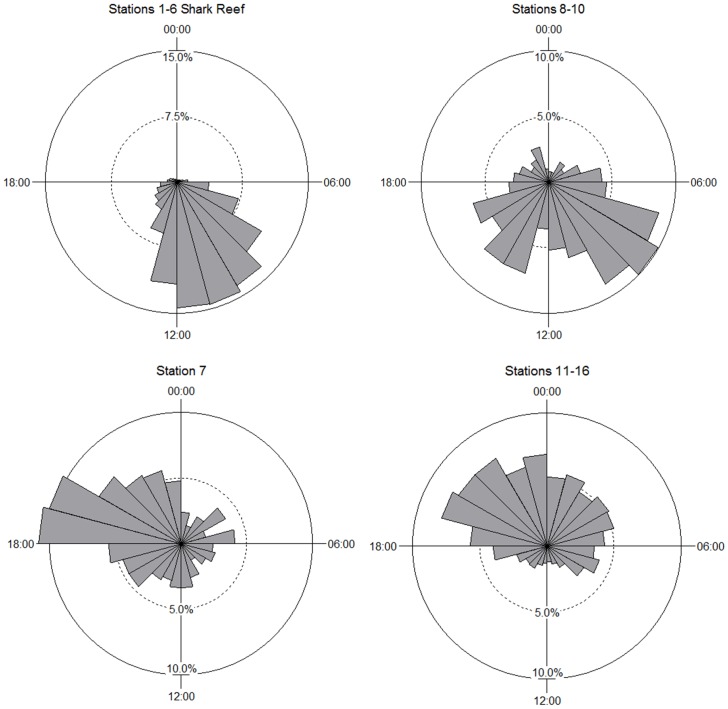
Circular plots showing the percentage of hours *C. leucas* were detected at grouped stations over the 24 h diel cycle. Sample sizes (i.e. number of hours): Shark Reef, n = 12,688; Station 7, n = 438; Stations 8–10, n = 514; Stations 11–16, n = 1,466.

A more detailed analysis of area use comparing grouped stations (Shark Reef 1–6, Station 7, Stations 8–10 and 11–16) shows that *C. leucas* displayed temporal differences in the use of the areas within the array (Chi-square tests, p<0.001 for all comparisons). The general pattern is for sharks to occur at Shark Reef, but also to move to neighbouring reefs (Stations 8–10) during the day (Figs. 1 and 5). Between 18:00–00:00 *C. leucas* peak in area use around Station 7 before moving further afield for the remainder of the nocturnal period, which includes Stations 11–16 (Fig. 5).

### Site fidelity

Site fidelity indexes SFI_a_ of *C. leucas* monitored for >10 days varied among individuals and, with one exception (ID 32), remained the same or decreased from Array to 1_9-12_ (Table 3). SFI_a_ values decreased with increasing number of days transmitters were attached to individual *C. leucas* for Array (*y* = −0.0007*x*+0.7449, R^2^ = 0.4, p<0.05), SR (*y* = −0.0007*x*+0.6661, R^2^ = 0.02, p<0.05) and 1 (*y* = −0.0006*x*+0.6013, R^2^ = 0.37, p<0.05), but not 1_9-12_ (*y* = −0.0004*x*+0.4819, R^2^ = 0.25, p>0.05) (Fig. S4). The majority of *C. leucas* had site fidelity indexes >0.5 for Array, SR, and 1 (80.6%, 72.2% and 65.5%, respectively), whereas 58.6% of *C. leucas* had SFI_a_ values ≤0.5 for 1_9-12_ (Table 3). Site fidelity indexes SFI_a_ 1_9-12_ did not differ between feeding and non-feeding days (Mann–Whitney *U*-test, p>0.05; Table 3).

**Figure 6:**
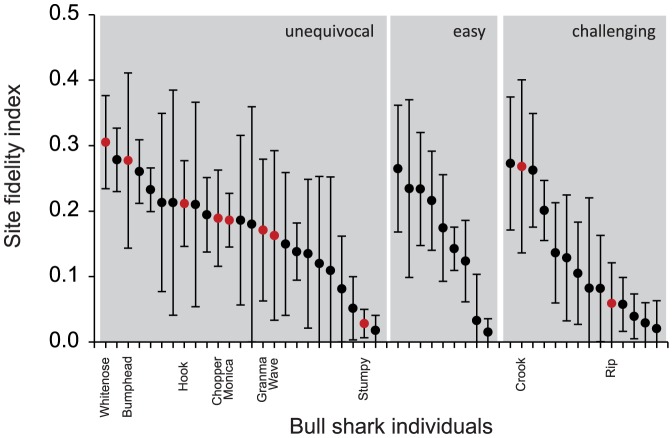
Site fidelity indexes (SFI_v_) determined from direct observation of visually identifiable *C. leucas* at the Shark Reef Marine Reserve between 2004 and 2011. Mean SFI_v_ values of 25 ‘unequivocal’, 9 ‘easy’ and 14 ‘challenging’ to identify *C. leucas* individuals (see text for details). SD  =  variation between years. Red dots denote individuals for which also acoustic monitoring data are available (see Table 3). Refer to Table S1 in [18] for description of natural marks of individuals.

**Figure 7:**
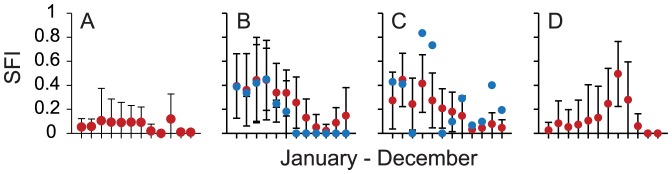
Examples of intraspecific variation of *C. leucas* monthly encounter rates. Representative examples of mean (±SD) monthly SFI_v_ values from (A) Rip, (B) Crook, (C) Hook and (D) Long John. Red dots denote SFI_v_ values; blue dots denote SFI_a_ 1_9-12_ values. Standard deviations represent variation between years (SFI_v_ = 2004–2011; SFI_a_ = monitoring period). Refer to Table S1 in [18] for description of natural marks of individuals.

Mean site fidelity indexes SFI_v_ for 48 individual *C. leucas* that could be visually identified based on external markings and pigmentation ranged from 0.02–0.31 (Fig. 6, Table S1), and did not differ between *C. leucas* individuals that were ‘unequivocal’, ‘easy’ or ‘challenging’ to identify (ANOVA, p = 0.236). Whereas mean monthly SFI_v_ values of some *C. leucas* individuals were within a relatively narrow range throughout a calendar year (Fig. 7A), others were encountered at higher rates only or not at all in certain months of the year (Fig. 7D, Table S2). However, a general pattern found was that most individuals were encountered less often in the second half of a calendar year (Fig. 7B and C, Table S2).

Site fidelity indexes of *C. leucas* individuals determined by focal observation at the feeding site (SFI_v_; n = 48 individuals) were lower compared to SFI_a_ 1_9-12_ values (n = 29 transmitters) determined by acoustic monitoring (mean ± SD = 0.16±0.08 vs. mean±SD = 0.45±0.2; Mann–Whitney *U*-test, p<0.001).

### Intraspecific Variation – Examples

#### Between Individuals

**Figure 8:**
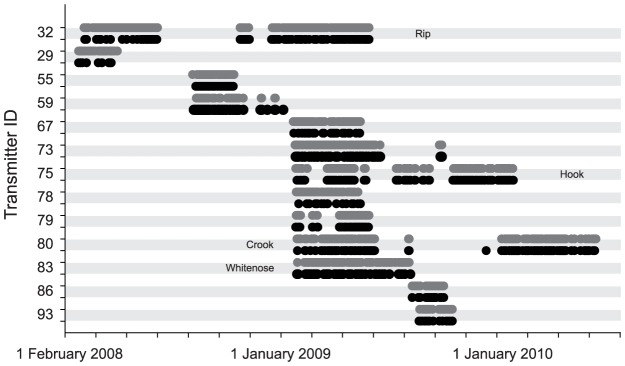
Timeline of the daily detections of *C. leucas* monitored for >50 days. Grey dots denote detections at Shark Reef (Stations 1–6) and black dots detections off Shark Reef (Stations 7–16).

The number of times *C. leucas* tagged for >50 days returned to the array and/or Shark Reef after being absent for periods of few days to several months (Fig. 8) ranged from 5–38 (mean ± SD = 17.9±11.4) and 7–44 (mean ± SD = 21.1±13.8), respectively (Table 4). Three female individuals (IDs 32, 73, 80) were not detected at Station 1 between 09:00 and 12:00 for >100 consecutive days (maximum  = 210 days, ID 80), all in the second half of a calendar year (Table 4). With the exception of *C. leucas* ID 80, if sharks were absent from Shark Reef and/or the feeding site for longer time periods, they were also not detected outside the protected area (Fig. 8, Table 4).

**Table 4:** number of consecutive days *C. leucas* monitored for >50 days were not detected in the array (Stations 1–16), at Shark Reef (Stations 1–6) and at Station 1 between 09:00 and 12:00, and number of returns to the receiver array and Shark Reef.

Transmitter ID	Number of days monitored (moy)	Maximum number of consecutive days (moy) not detected in/at:			Number of returns to:	
		Array	SR	1_9-12_	Array	SR
29	63 (February–April)	4	4	8	9	9
32	462 (all)	133	133	133^a^ (July–November)	28	40
55	68 (August–November)	4	6	6^a^	6	7
59	145 (August–January)	27	28	28	16	14
67	109 (February–May)	8	8	8	16	20
73	239 (February–October)	95	95	103 (June–September)	19	18
75	355 (all)	48	49	51^a^	28	40
78	105 (February–May)	4	4	6^a^	18	17
79	119 (February–June)	34	35	35	5	9
80	486 (all)	124	149	210^a^ (June–January)	36	44
83	184 (February–August)	9	10	10	38	36
86	51 (August–October)	8	8	8	6	9
93	57 (August–October)	8	9	20	8	10

moy  =  month(s) of the year.

a Minimum number of days because period not entirely covered by Station 1 (see Table 2)

Three female *C. leucas* (Rip ID 32, Hook ID 75 and Crook ID 80) were monitored with external tags for periods >1 year (Figs. 8 and S2, Table 1). Hook, an individual unequivocal to visually identify and Crook, an individual challenging to identify, have both been regular visitors to the feeding site with overall SFI_v_ values of 0.21 and 0.27, respectively (Fig. 6, Table S1). Although lower, these are values in a similar range compared to the respective values determined by acoustic monitoring (Table 3). *Carcharhinus leucas* Rip (ID 32), like Crook rated challenging to visually identify by the observers, on the other hand had a much larger SFI_a_ value (0.48; Table 3) compared to the overall SFI_v_ value (0.06) determined from direct observation at the feeding site (Fig. 6, Table S1). With tags attached externally, however, all three *C. leucas* individuals were, if detected at Station 1 between 09:00 and 12:00 on feeding days, visually confirmed to be present with likelihoods in a similar range (Rip = 40%, Crook = 45%, Hook = 52%).

**Figure 9:**
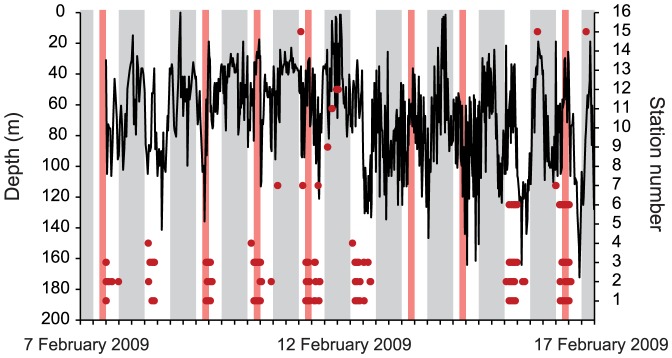
Time-depth series of satellite tagged *C. leucas* Rip. In addition to acoustic tag ID 32, the female shark was tagged with a PSAT (standard rate X-Tag; for tag specifications see F14 in [20]) for 9 days in February 2009. Red dots denote acoustic detections at Shark Reef (Stations 1–6) and off Shark Reef (Stations 7–16). Grey shaded areas denote night (18:00–06:00); light red shaded areas denote days and time (09:00–12:00) of feeding.

All three female *C. leucas* were tagged in the first quarter of a calendar year (Table 1), but showed different residency patterns during the monitoring periods. Both Crook and Rip were regularly detected within the receiver array for the first few months after tagging and then left the receiver array for several months in the second half of the calendar year (Fig. 8, Table 4). After leaving in June 2008, Rip was not detected for 133 consecutive days (Table 4) before returning to the array and occasionally Shark Reef in November for two weeks. After being again not detected in the receiver array for 35 consecutive days the shark resumed a residency pattern of being detected in the array and at Shark Reef for several consecutive days or weeks, interspersed with periods of absence of several consecutive days until the end of the monitoring period in June 2009 (Fig. 8). This pattern and the associated vertical niche are exemplified by data collected when Rip was double-tagged with acoustic transmitter ID 32 and a PSAT between 7 and 16 February 2009. During this time period, the shark was detected at receivers mostly during daytime and at Station 1 between 09:00 and 12:00 on every day except for 13 and 14 February when it was not recorded in the receiver array (Fig. 9). Mean swimming depth on days Rip was detected in the array was lower compared to depths recorded on 13 and 14 February (mean ± SD = 64.4±30.6 m vs. mean ± SD = 76.3±28.5 m; Mann–Whitney *U*-test, p<0.001; Fig. 9). Despite being detected multiple times each day at Shark Reef stations including Station 1 during the time feeding took place at Shark Reef (n = 6 days), Rip was visually confirmed to be present on only two of these days (9 and 10 February).

Crook, the overall longest tagged *C. leucas* (Table 1), left the receiver array in June 2009. With the exception of two consecutive days in August when it was detected at Shark Reef, but not the feeding station in the morning hours, and one day in December when Crook was detected off Shark Reef, this individual was not detected in the receiver array until January 2010. From then on, this individual was again detected at Shark Reef on a regular basis until the end of the monitoring period in June 2010 (Fig. 8, Table 4).

*Carcharhinus leucas* Hook was monitored for 355 days between February 2009 and January 2010. The residency pattern of this individual can be characterized by blocks of detection periods of several weeks during which Hook was detected on few to several consecutive days interspersed with periods of absence from the receiver array of similar lengths (Fig. 8). A similar residency pattern, although shorter in duration, was recorded for the longest monitored male *C. leucas* individual (Whitenose, ID 83), which was tagged for 184 days between February and August 2009 (Fig. 8, Tables 1 and 3).

#### Within Individuals

Female *C. leucas* Monica was tagged a total of four times in 2007 and 2008 (all stomach tags) with two monitoring periods >10 days (IDs 12 and 50; Table 1). This individual has been a regular visitor to the feeding site since 2004 with an overall SFI_v_ value of 0.19 (Fig. 6, Table S1). Tag ID 12 was fed to Monica on 4 January 2007 and the shark was monitored until 15 January 2007 (Table 1). During this time period, Monica was detected within the receiver array, at Shark Reef and, with the exception of the last day of the monitoring period, at Station 1 between 09:00 and 12:00 on every single day (Table 3). On all the days Monica was visually identified at the feeding site (n = 7), the presence of this shark was confirmed by acoustic detections at Station 1 between 09:00 and 12:00. The second tag (ID 50) was fed to Monica on 25 July 2008 and the shark was monitored until 7 August 2008 (Table 1). Contrary to the previous monitoring period, Monica was not detected by any of the receivers except for the last day of the monitoring period. On this day when feeding took place at Shark Reef, Monica was first detected at Station 11 just after noon and then again between 17:00 and 17:30 at Shark Reef (Station 2).

### Stomach Temperatures and Depths

Mean stomach temperatures recorded by transmitters fed to *C. leucas* individuals ranged from 26.54–28.58 °C (Table S3). Stomach temperatures recorded between June and August 2008 were lower compared to those recorded in February and March 2009 (mean ± SD = 26.61±0.27 °C vs. mean±SD = 27.92±0.82°C; Mann–Whitney *U*-test, p<0.001), reflecting the cooler/warmer ambient water temperatures in austral summer and winter, respectively (Fig. S5). Mean swimming depths recorded by transmitters attached externally to *C. leucas* individuals and logged by receivers ranged from 31.3–39.6 m (Table S3).

## Discussion

Aggregations of single or multiple species of sharks at small spatial scales are known to occur both naturally [25]–[28] and at ecotourism provisioning sites [15], [29], but still relatively little is known from only a few species about their residency and movements patterns in the latter context [13]–[16]. Our results contribute to filling this knowledge gap. Overall site fidelity, presence/absence and movement patterns of *C. leucas* were similar on provisioned and non-provisioned days. Moreover, our results from food-provisioned adult *C. leucas* from Fiji are generally in line with similar results from other, comparable, provisioned and not, shark species, and also non-provisioned *C. leucas* as shown by the following discussion.

The little information currently available on adult *C. leucas* movement behaviour indicates that individuals primarily use shallow marine habitats close to coasts and have generally small activity spaces, staying in limited areas over long periods without showing pronounced large-scale movements [30], [31]. Such behaviour was also found for electronically tagged *C. leucas* at the Shark Reef Marine Reserve ([20]; this study). Similar to other shark species associated with coral reefs and/or provisioning sites (e.g. [13], [32]), individual *C. leucas* also were absent from the study area for several weeks to months and left the area on larger scale before returning to the feeding site ([20]; this study). Our results clearly show that *C. leucas* individuals are not permanent residents at the Shark Reef Marine Reserve, but use the broader coastal area at different temporal scales on the southern coast of Viti Levu.

The use of restricted areas or resting in a core area during the day followed by dispersal at night to cover greater areas, presumably in the context of increased foraging activity, has been observed in a number of both food-provisioned and non-provisioned elasmobranch species [13], [26], [28], [33]–[35]. We found a similar pattern, namely that *C. leucas* were using Shark Reef throughout the day and dispersing over the entire array at night. Interestingly, only at night *C. leucas* moved to Station 12, the only estuary location covered by the array (Fig. 1) which might indicate that sharks forage in the Navua River mouth or even up river at night. *Carcharhinus leucas* are known to increase their feeding activity during nocturnal hours and to be associated with riverine/estuarine habitats [36]–[38].

Despite year-round availability of food, *C. leucas* were not permanently attracted to the Shark Reef Marine Reserve. Individual adult *C. leucas* showed weak to strong site fidelity to the receiver array (range 0.14–1; Table 3), a range similar to what has been found for other species [39], [40]. Lower site fidelity values, both on feeding and non-feeding days, were recorded for Station 1, indicating that individuals do not necessarily come to the feeding site even on days when present in the area and food is on offer. This, together with the anecdotal observations of very few *C. leucas* on non-feeding days and sharks leaving the feeding site as soon as feeding stops, indicates that *C. leucas* avoid the area when humans are present, and hence food provisioning is essential to elicit human-oriented *C. leucas* behaviour. As such and *sensu* Knight [1], *C. leucas* can be considered ‘typical wild animals’ that are generally human-averse. Their behavioural response to humans is in stark contrast to some coastal teleosts that, after learning to associate food with human presence, lose fear and encircle people in the water even when food is not provided in fish-feeding areas [41], [42]. At the same time, our finding that site fidelity indexes determined from direct diver observation were generally smaller compared to such values determined from acoustic monitoring, exemplifies the likelihood of bias in the collection of presence/absence, abundance or behavioural data of mobile fish using underwater visual census and observation techniques.

Both from visual and acoustic monitoring, detections at the feeding site were highest in the first half of a calendar year for most *C. leucas*, although some individuals visit Shark Reef year-round ([18]; this study). Such seasonal patterns and intraspecific variability in the level of site visitation/attendance have been observed in other species too, both at sites where sharks naturally aggregate [25], [26], [32], [43] and to where they are attracted for tourism purposes [15], [16], [29], and have generally been suggested to relate to reproductive activity. Our results allow us to conclude that *C. leucas* individuals do not simply stay away from the feeding site or out of sight at certain times of the year [18], but leave the area on larger scale. However, it remains unknown where exactly reproductive activity of Fijian *C. leucas* takes place.

Over the past few years we have studied *C. leucas* visiting the Shark Reef Marine Reserve using satellite [20] and acoustic telemetry (this study) as well as direct diver observations ([18]; this study). The picture that is emerging can be summarized as follows: Food provisioning by means of hand-feeding appears to congregate, at least at certain times of the year, large numbers of *C. leucas* in waters around Shark Reef on the southern coast of Viti Levu [18]. If present in the area, sharks may come to the Shark Reef Marine Reserve regardless of feeding or non-feeding days, but remain for longer periods of time on feeding days. Individual *C. leucas* show varying degrees of site fidelity to the feeding site, Shark Reef and neighbouring reefs. The overall diel patterns in local-scale movement are for *C. leucas* to use the area around the feeding site in the morning before spreading out over Shark Reef throughout the day and dispersing over the entire array, including crossing the Beqa Channel, more at night. Both focal observation and acoustic monitoring show that *C. leucas* intermittently leave the area for a few consecutive days throughout the year, and longer time periods generally at the end of the calendar year, before returning to the feeding site. In summary, our results indicate that *C. leucas* respond to the food on offer when encountering it, but the feeding operation does not appear to drive their long-term movements and the sharks are not strongly conditioned as otherwise they would be expected to be present at almost every feed.

In conclusion, our results and the still few studies that looked at the behavioural response of sharks to food provisioning all indicate that residency patterns and site fidelity to long-term shark provisioning sites are species specific and that intraspecific variation exists. Furthermore, evidence is accumulating that chumming and food provisioning are unlikely to fundamentally change movement patterns at large spatial and temporal scales, and seem to only have a minor impact on the behaviour of large predatory sharks [14], [16], [44]; hence, the creation of behavioural effects at the ecosystem level seems unlikely [44]. It is further worth noting that sharks that were both visually observed and tagged in this study were individuals that have a higher propensity for showing behavioural responses to provisioning. We found that *C. leucas* do not appear to be strongly conditioned to the provisioning tourism and also exhibited diver avoidance. However, the sharks monitored in this study are biased to being individuals ‘more likely’ or ‘more comfortable’ to be observed or tagged. Thus, it stands to reason that the overall impacts of provisioning tourism on the *C. leucas* population as a whole is even less.

But despite the indication that levels of residency and diel activity and behaviour as well as local-scale movement patterns found for *C. leucas* in this and previous studies [20] can be regarded as ‘normal’, it is possible that hand-feeding sharks at the Shark Reef Marine Reserve for more than 10 years has been attracting *C. leucas* to the area, and that individual sharks visit Shark Reef more often and/or spend more time in the area. This may raise concerns about increased susceptibility to local fishing operations [13]. However, the feeding operation we looked at here is closely linked to a local marine conservation project which protects all sharks in the Shark Reef Marine Reserve and adjacent coastal areas [4]. Marine reserves are increasingly being proposed for elasmobranch conservation [28], [34], [45], [46], but only a few studies have used movement analysis to test the effectiveness of marine reserves in protecting elasmobranchs (e.g. [34], [43], [45], [47]). As such, the Shark Reef Marine Reserve is another example of how shark provisioning tourism can be an effective strategy that can contribute to apex predator conservation, this time for *C. leucas* [16].

## Supporting Information

Figure S1 **Mean (± SD) number of** ***C. leucas*** **counted on the first dive at 30 m (n = 1,270 dives) and on the second dive of the day at 16 m (n = 1,184 dives) between 2003 and 2011.** For count methodology see [18].
(PDF)

Figure S2 **Monitoring periods of 79 acoustically tagged** ***C. leucas*** **(** **Table 1** **) in the receiver array.** For those transmitters that were attached to sharks 31 December/1 January (IDs 32, 59, 75, 80), the day of tag attachment is indicated with a red square and the end of the monitoring period with a green square.
(PDF)

Figure S3 **Hourly percentage of detections of** ***C. leucas*** **in the receiver array.** Tagged *C. leucas* were detected (total number of detections = 114,282) at Shark Reef receivers (white dots = Stations 1–6, 106,995 detections; grey dots  =  Station 1 only, 42,595 detections) from early morning to early afternoon, particularly between 09:00 and 12:00. Black dots denote receivers off Shark Reef (Stations 7–16, 7,287 detections). SD = variation between receivers. The red shaded area denotes the time of the day when feeding occurred at the Shark Reef Marine Reserve.
(PDF)

Figure S4 **Regression analysis was used to evaluate whether or not SFI_a_ values decreased with increasing number of days transmitters were attached to individual** ***C. leucas*** **.** SFI_a_ values decreased for Array (black dots; *y* = −0.0007*x*+0.7449, R^2^ = 0.4, p<0.05), SR (grey dots; *y* = −0.0007*x*+0.6661, R^2^ = 0.02, p<0.05) and 1 (white dots; *y* = −0.0006*x*+0.6013, R^2^ = 0.37, p<0.05), but no trend was detected for 1_9–12_ (red dots; *y* = −0.0004*x*+0.4819, R^2^ = 0.25, p>0.05).
(PDF)

Figure S5 **Water temperature in the Shark Reef Marine Reserve was recorded with UTBI-001 TidbiT v2 data loggers.** From June to August 2008, water temperature was recorded by one logger placed at 10 m (recording interval 5 min). Between February and March 2009, water temperature was calculated (mean) from two data loggers (recording intervals 60 min) placed at 10 m and 30 m. Black horizontal lines are mean water temperatures and blue horizontal lines are mean stomach temperatures from *C. leucas* individuals tagged during the respective time period (n = 3 in 2008, n = 9 in 2009; see Table 1 for details).
(PDF)

Table S1 **Annual site fidelity indexes (SFIa) determined from visual monitoring of** ***C. leucas*** **at the feeding site in the Shark Reef Marine Reserve between 2004 and 2011.** Refer to Table S1 in [18] for description of natural marks of individuals.
(PDF)

Table S2 **Mean monthly SFIv values (SD  =  variation between years) from 48** ***C. leucas*** **individuals visually monitored at the Shark Reef Marine Reserve between 2004 and 2011.** Refer to Table S1 in [18] for description of natural marks of individuals.
(PDF)

Table S3 **Minimum, maximum and mean (±SD) stomach temperatures (°C) and depths (m) recorded by transmitters with temperature or pressure sensors.**
(PDF)
