# Supplementary figures and images for: Opportunistic Visitors: Long-Term Behavioural Response of Bull Sharks to Food Provisioning in Fiji
Source: PLoS One. 2013 Mar 13;8(3):e58522. doi: 10.1371/journal.pone.0058522 (PMC3596312; doi:10.1371/journal.pone.0058522)

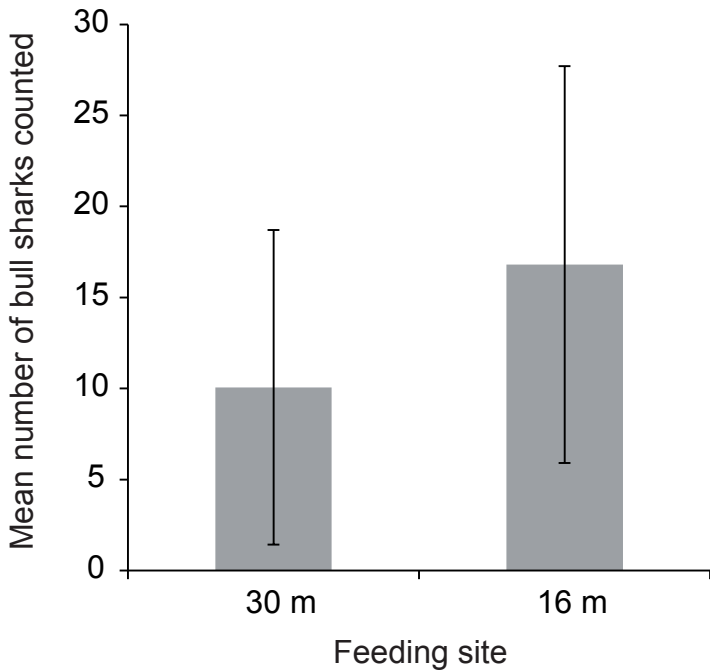

Supplement: Figure S1 — Mean (± SD) number of C. leucas counted on the first dive at 30 m (n = 1,270 dives) and on the second dive of the day at 16 m (n = 1,184 dives) between 2003 and 2011. For count methodology see [18]. (PDF) [file pone.0058522.s001.pdf]

Transmitter ID

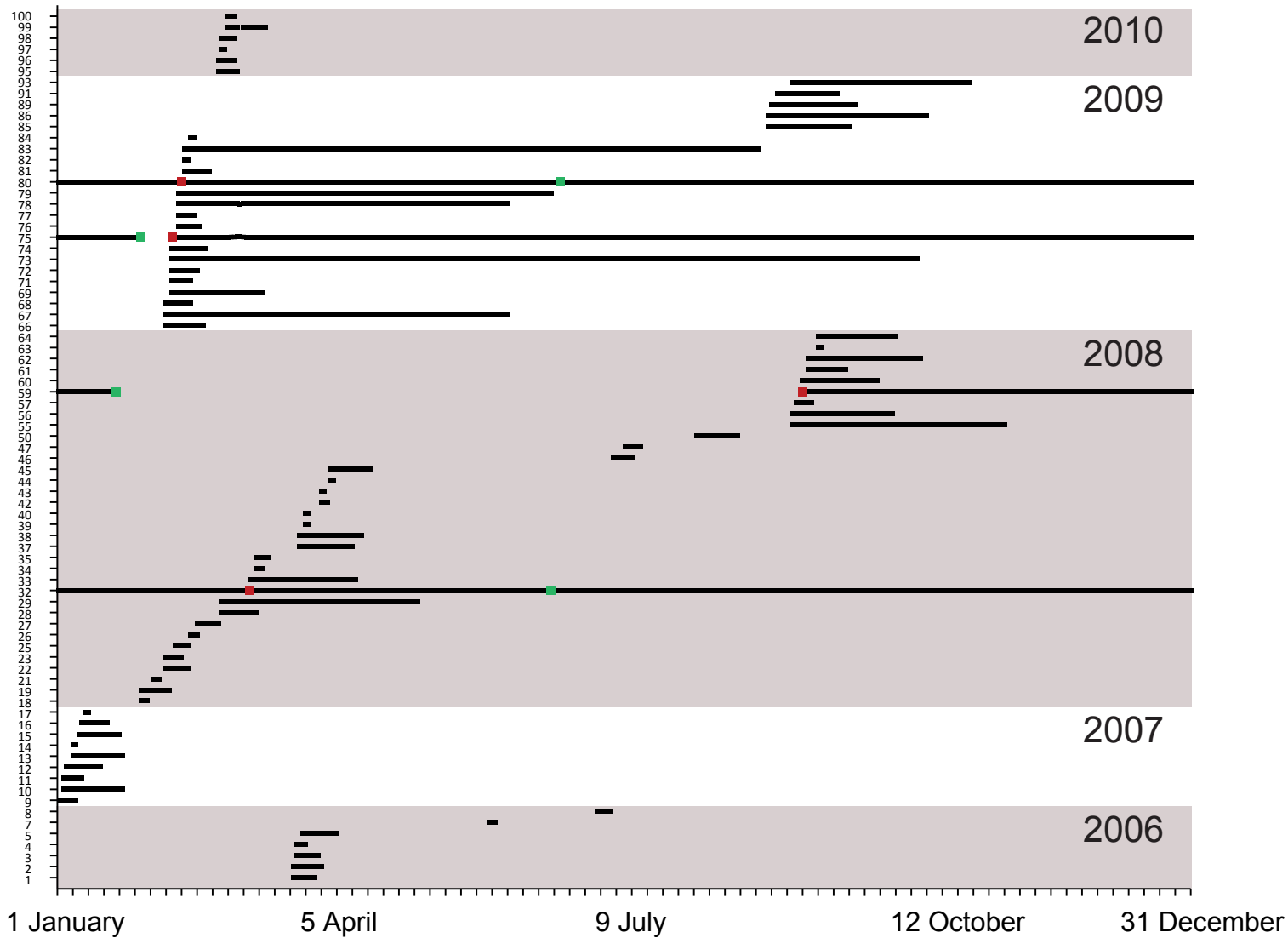

Supplement: Figure S2 — Monitoring periods of 79 acoustically tagged C. leucas ( Table 1 ) in the receiver array. For those transmitters that were attached to sharks 31 December/1 January (IDs 32, 59, 75, 80), the day of tag attachment is indicated with a red square and the end of the monitoring period with a green square. (PDF) [file pone.0058522.s002.pdf]

Percentage detections

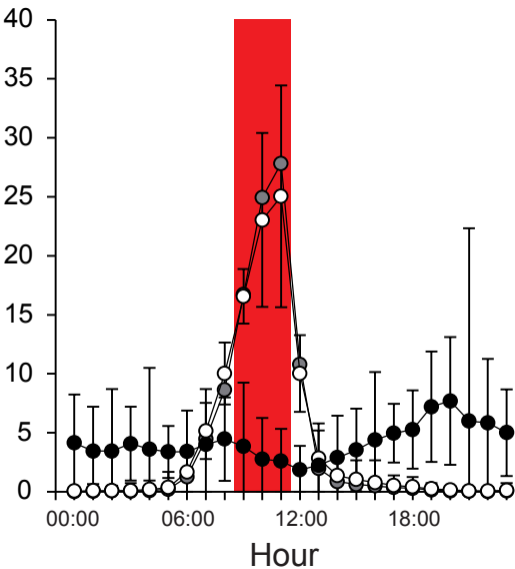

Supplement: Figure S3 — Hourly percentage of detections of C. leucas in the receiver array. Tagged C. leucas were detected (total number of detections = 114,282) at Shark Reef receivers (white dots = Stations 1–6, 106,995 detections; grey dots = Station 1 only, 42,595 detections) from early morning to early afternoon, particularly between 09:00 and 12:00. Black dots denote receivers off Shark Reef (Stations 7–16, 7,287 detections). SD = variation between receivers. The red shaded area denotes the time of the day when feeding occurred at the Shark Reef Marine Reserve. (PDF) [file pone.0058522.s003.pdf]

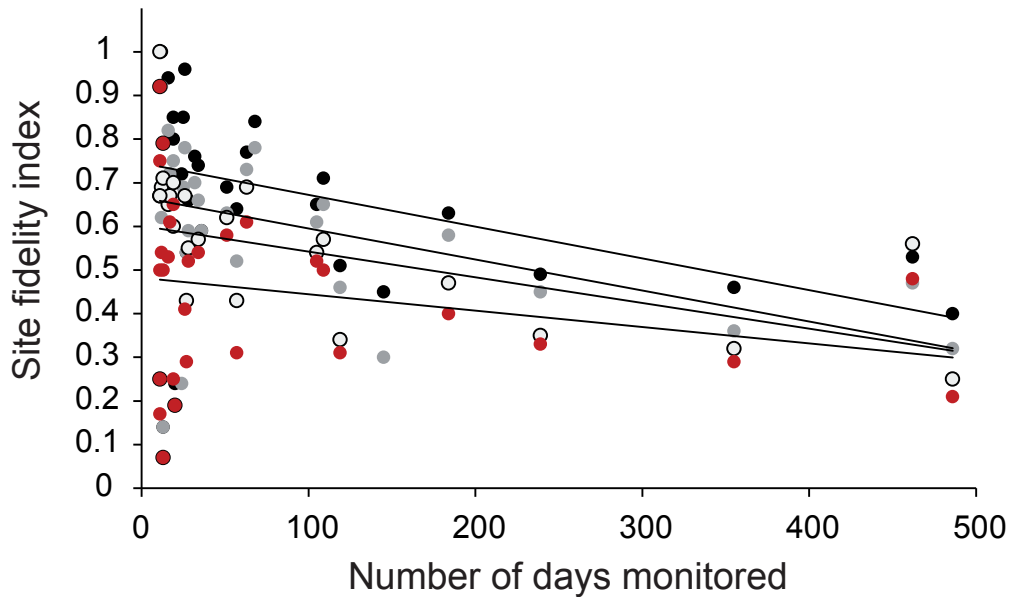

Supplement: Figure S4 — Regression analysis was used to evaluate whether or not SFIa values decreased with increasing number of days transmitters were attached to individual C. leucas . SFIa values decreased for Array (black dots; y = −0.0007x+0.7449, R2 = 0.4, p<0.05), SR (grey dots; y = −0.0007x+0.6661, R2 = 0.02, p<0.05) and 1 (white dots; y = −0.0006x+0.6013, R2 = 0.37, p<0.05), but no trend was detected for 19–12 (red dots; y = −0.0004x+0.4819, R2 = 0.25, p>0.05). (PDF) [file pone.0058522.s004.pdf]

Temperature °C

31  
30  
29  
28  
27  
26  
25  
24

28 June - 7 August 2008

11 February - 7 March 2009

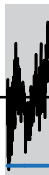

Supplement: Figure S5 — Water temperature in the Shark Reef Marine Reserve was recorded with UTBI-001 TidbiT v2 data loggers. From June to August 2008, water temperature was recorded by one logger placed at 10 m (recording interval 5 min). Between February and March 2009, water temperature was calculated (mean) from two data loggers (recording intervals 60 min) placed at 10 m and 30 m. Black horizontal lines are mean water temperatures and blue horizontal lines are mean stomach temperatures from C. leucas individuals tagged during the respective time period (n = 3 in 2008, n = 9 in 2009; see Table 1 for details). (PDF) [file pone.0058522.s005.pdf]
