# Supplementary material for: Opportunistic Visitors: Long-Term Behavioural Response of Bull Sharks to Food Provisioning in Fiji
Source: PLoS One. 2013 Mar 13;8(3):e58522. doi: 10.1371/journal.pone.0058522 (PMC3596312; doi:10.1371/journal.pone.0058522)
Supplement: Table S1 — Annual site fidelity indexes (SFIa) determined from visual monitoring of C. leucas at the feeding site in the Shark Reef Marine Reserve between 2004 and 2011. Refer to Table S1 in [18] for description of natural marks of individuals. (PDF) [file pone.0058522.s006.pdf]

Table S1. Annual site fidelity indexes (SFI_a) determined from visual monitoring of *C. leucas* at the feeding site in the Shark Reef Marine Reserve between 2004 and 2011. Refer to Table S1 in [18] for description of natural marks of individuals.

	**2004**	**2005**	**2006**	**2007**	**2008**	**2009**	**2010**	**2011**	**Mean (± SD)**
Annie	0.21	0.01	0	0.02	0	0	0.01	0.02	0.03 (0.07)
Bum	0.24	0.27	0.17	0.25	0.25	0.20	0.24	0.23	0.23 (0.03)
Cilla	0.03	0.01	0	0.07	0	0.02	0.01	0	0.02 (0.02)
Crook	0.48	0.20	0.16	0.24	0.23	0.22	0.14	0.47	0.27 (0.13)
Crush	0.1	0.07	0.05	0.02	0	0	0.04	0.14	0.05 (0.05)
Hook	0.32	0.20	0.27	0.19	0.14	0.15	0.16	0.26	0.21 (0.07)
Kinky	0.30	0.22	0.09	0.17	0.08	0.10	0.06	0	0.13 (0.10)
Stumpy	0.04	0.04	0.07	0.03	0.01	0.01	0.03	0	0.03 (0.02)
Long John	0.08	0	0.07	0.18	0.14	0.16	0.07	0.38	0.14 (0.11)
Whitenose	0.39	0.41	0.27	0.34	0.23	0.22	0.26	0.32	0.31 (0.07)
Flop	0.17	0.42	0.20	0.20	0.24	0.24	0.23	0.42	0.26 (0.10)
Granma	0.02	0.32	0.30	0.10	0.15	0.14	0.10	0.25	0.17 (0.12)
Monica	0.21	0.17	0.17	0.14	0.16	0.16	0.24	0.25	0.19 (0.04)
Rip	0.03	0.01	0.02	0.03	0.05	0.16	0.16	0.02	0.06 (0.06)
Blackbeard	0.12	0.01	0	0	0.01	0	0.03	0	0.02 (0.04)
Chopper	0.05	0.11	0.24	0.19	0.21	0.21	0.24	0.27	0.19 (0.07)
Grin	0.42	0.06	0	0.02	0.23	0.04	0.03	0.08	0.11 (0.14)
Second				0.19	0.10	0.02	0.22	0.14	0.14 (0.08)
Hotlips				0.27	0.20	0.22	0.18	0.14	0.20 (0.05)
Chica				0.21	0.06	0.23	0.23	0.44	0.23 (0.14)
Valerie				0.38	0.26	0.11	0.11	0.08	0.19 (0.13)
Detour				0.24	0.09	0.23	0.29	0.31	0.23 (0.09)
Bumphead				0.50	0.27	0.22	0.14	0.25	0.28 (0.13)
Topsail				0.17	0.11	0.10	0.27	0.42	0.21 (0.14)
Rusty					0.08	0.19	0.05	0	0.08 (0.08)
Line					0.07	0.04	0.01	0	0.03 (0.03)
Scar					0.11	0.07	0.01	0.05	0.06 (0.04)
Curly					0.02	0.14	0.27	0.42	0.21 (0.17)
Maite						0.08	0.22	0.22	0.17 (0.08)
Lee						0.30	0.22	0.31	0.28 (0.05)
Sierra						0.06	0.06	0	0.04 (0.03)
Blunt						0.21	0.13	0.24	0.19 (0.06)
Marlen						0.14	0.11	0.17	0.14 (0.03)
Pointer						0.21	0.26	0.31	0.26 (0.05)
Sickle						0.03	0.26	0.33	0.21 (0.16)
Trailer						0.18	0.17	0.30	0.22 (0.08)
Twist						0.15	0.15	0.02	0.11 (0.08)
Moana						0.06	0.13	0.18	0.12 (0.06)
Tip						0.16	0.30	0.36	0.27 (0.10)
Brenda						0.03	0.13	0.38	0.18 (0.18)
Big Mama						0.04	0	0.01	0.02 (0.02)
Trevally						0.09	0.16	0.17	0.14 (0.04)
Nani						0.03	0.17	0.25	0.15 (0.11)
Lill						0.24	0.01	0	0.08 (0.14)
Wave						0.03	0.17	0.29	0.16 (0.13)
Gill						0.03	0.04	0.17	0.08 (0.08)
Junior						0.03	0.06	0.27	0.12 (0.13)
Naughtylus						0.33	0.17	0.29	0.26 (0.09)
