# Supplementary material for: Opportunistic Visitors: Long-Term Behavioural Response of Bull Sharks to Food Provisioning in Fiji
Source: PLoS One. 2013 Mar 13;8(3):e58522. doi: 10.1371/journal.pone.0058522 (PMC3596312; doi:10.1371/journal.pone.0058522)
Supplement: Table S2 — Mean monthly SFIv values (SD = variation between years) from 48 C. leucas individuals visually monitored at the Shark Reef Marine Reserve between 2004 and 2011. Refer to Table S1 in [18] for description of natural marks of individuals. (PDF) [file pone.0058522.s007.pdf]

Table S2. Mean monthly SFI_v values (SD = variation between years) from 48 *C. leucas* individuals visually monitored at the Shark Reef Marine Reserve between 2004 and 2011. Refer to Table S1 in [18] for description of natural marks of individuals.

	Jan	Feb	Mar	Apr	May	Jun	Jul	Aug	Sep	Oct	Nov	Dec
Annie	0.05 (0.13)	0.01 (0.02)	0.08 (0.19)	0.05 (0.12)	0.05 (0.13)	0.05 (0.11)	0.01 (0.03)	0.01 (0.04)	0.02 (0.05)	0.05 (0.13)	0.03 (0.08)	0 (0)
Bum	0.36 (0.12)	0.37 (0.27)	0.43 (0.17)	0.15 (0.12)	0.28 (0.13)	0.30 (0.19)	0.19 (0.18)	0.10 (0.07)	0.09 (0.10)	0.03 (0.06)	0.07 (0.10)	0.22 (0.23)
Cilla	0.01 (0.03)	0.01 (0.03)	0.02 (0.05)	0 (0)	0.02 (0.06)	0.01 (0.02)	0.01 (0.03)	0.04 (0.07)	0.01 (0.03)	0.05 (0.13)	0 (0)	0.07 (0.19)
Crook	0.39 (0.27)	0.36 (0.30)	0.44 (0.36)	0.44 (0.33)	0.34 (0.24)	0.34 (0.19)	0.26 (0.21)	0.13 (0.16)	0.05 (0.11)	0.02 (0.05)	0.09 (0.13)	0.15 (0.23)
Crush	0.14 (0.18)	0.04 (0.05)	0.05 (0.09)	0 (0)	0.06 (0.08)	0.05 (0.10)	0.07 (0.10)	0.04 (0.07)	0.03 (0.06)	0.09 (0.11)	0.02 (0.03)	0.09 (0.22)
Hook	0.27 (0.24)	0.44 (0.22)	0.24 (0.22)	0.41 (0.24)	0.27 (0.23)	0.21 (0.16)	0.18 (0.16)	0.15 (0.17)	0.03 (0.04)	0.04 (0.06)	0.08 (0.09)	0.05 (0.07)
Kinky	0.05 (0.10)	0 (0)	0.02 (0.03)	0.21 (0.20)	0.30 (0.23)	0.28 (0.24)	0.23 (0.24)	0.16 (0.16)	0.14 (0.19)	0.02 (0.06)	0 (0)	0 (0)
Stumpy	0.16 (0.37)	0.05 (0.07)	0.07 (0.16)	0.01 (0.02)	0.01 (0.04)	0.03 (0.05)	0.03 (0.06)	0.02 (0.06)	0 (0)	0.02 (0.04)	0 (0)	0.04 (0.12)
Long John	0.03 (0.07)	0.08 (0.19)	0.05 (0.15)	0.07 (0.20)	0.11 (0.30)	0.13 (0.26)	0.25 (0.29)	0.49 (0.27)	0.28 (0.32)	0.06 (0.10)	0 (0)	0 (0)
Whitenose	0.41 (0.21)	0.74 (0.27)	0.56 (0.21)	0.49 (0.30)	0.33 (0.17)	0.31 (0.18)	0.22 (0.13)	0.18 (0.08)	0.10 (0.12)	0 (0)	0 (0)	0.04 (0.07)
Flop	0.46 (0.28)	0.42 (0.27)	0.51 (0.19)	0.32 (0.19)	0.36 (0.28)	0.29 (0.21)	0.15 (0.14)	0.11 (0.17)	0.03 (0.06)	0.04 (0.08)	0.05 (0.07)	0.21 (0.27)
Granma	0.22 (0.39)	0.26 (0.16)	0.16 (0.15)	0.16 (0.12)	0.21 (0.26)	0.16 (0.18)	0.23 (0.16)	0.17 (0.14)	0.12 (0.13)	0.11 (0.10)	0.11 (0.14)	0.17 (0.24)
Monica	0.30 (0.27)	0.19 (0.24)	0.15 (0.14)	0.29 (0.20)	0.27 (0.26)	0.19 (0.20)	0.25 (0.19)	0.17 (0.16)	0.01 (0.03)	0.04 (0.08)	0.03 (0.08)	0.15 (0.17)
Rip	0.05 (0.07)	0.06 (0.06)	0.11 (0.27)	0.09 (0.19)	0.09 (0.17)	0.09 (0.14)	0.09 (0.13)	0.02 (0.06)	0 (0)	0.12 (0.21)	0.01 (0.03)	0.01 (0.02)
Blackbeard	0 (0)	0.03 (0.06)	0.07 (0.16)	0.06 (0.12)	0.01 (0.02)	0.25 (0.71)	0.02 (0.05)	0.03 (0.07)	0.04 (0.11)	0 (0)	0 (0)	0 (0)
Chopper	0.33 (0.21)	0.41 (0.22)	0.41 (0.24)	0.17 (0.10)	0.19 (0.21)	0.22 (0.07)	0.23 (0.14)	0.12 (0.10)	0.09 (0.11)	0.06 (0.11)	0 (0)	0.07 (0.07)
Grin	0.12 (0.16)	0.12 (0.22)	0.20 (0.28)	0.07 (0.10)	0.16 (0.31)	0.25 (0.33)	0.14 (0.18)	0.07 (0.14)	0.04 (0.11)	0 (0)	0 (0)	0.01 (0.02)
Second	0.34 (0.35)	0.06 (0.06)	0.05 (0.07)	0.12 (0.09)	0.13 (0.13)	0.29 (0.27)	0.18 (0.14)	0.14 (0.25)	0.09 (0.11)	0.05 (0.11)	0 (0)	0.12 (0.15)
Hotlips	0.42 (0.31)	0.35 (0.33)	0.24 (0.25)	0.33 (0.24)	0.30 (0.12)	0.32 (0.22)	0.24 (0.14)	0.14 (0.14)	0.02 (0.04)	0.04 (0.08)	0 (0)	0.01 (0.03)
Chica	0.60 (0.32)	0.39 (0.34)	0.27 (0.21)	0.26 (0.16)	0.32 (0.29)	0.34 (0.35)	0.10 (0.12)	0.09 (0.10)	0 (0)	0 (0)	0.03 (0.06)	0.36 (0.19)
Valerie	0.10 (0.13)	0.16 (0.16)	0.13 (0.18)	0.24 (0.19)	0.37 (0.40)	0.30 (0.20)	0.21 (0.37)	0.07 (0.16)	0.04 (0.07)	0.05 (0.10)	0.07 (0.04)	0.21 (0.17)
Detour	0.20 (0.11)	0.26 (0.27)	0.38 (0.17)	0.48 (0.25)	0.47 (0.23)	0.37 (0.15)	0.23 (0.20)	0.12 (0.15)	0.07 (0.14)	0.04 (0.07)	0 (0)	0.04 (0.09)
Bumphead	0.10 (0.17)	0.19 (0.23)	0.35 (0.35)	0.47 (0.23)	0.56 (0.04)	0.33 (0.09)	0.16 (0.11)	0 (0)	0.03 (0.05)	0.12 (0.11)	0 (0)	0.20 (0.19)
Topsail	0.28 (0.27)	0.21 (0.25)	0.37 (0.43)	0.27 (0.23)	0.36 (0.35)	0.33 (0.28)	0.12 (0.12)	0.28 (0.16)	0.17 (0.23)	0.13 (0.14)	0 (0)	0.03 (0.07)
Rusty	0.06 (0.10)	0.13 (0.15)	0.12 (0.14)	0.12 (0.23)	0.15 (0.25)	0.12 (0.10)	0.13 (0.09)	0.03 (0.03)	0 (0)	0 (0)	0 (0)	0.10 (0.16)
Line	0.11 (0.19)	0.04 (0.06)	0.03 (0.04)	0 (0)	0 (0)	0.02 (0.03)	0.02 (0.03)	0 (0)	0 (0)	0 (0)	0 (0)	0.03 (0.06)
Scar	0.20 (0.18)	0.05 (0.10)	0.07 (0.05)	0.17 (0.25)	0.09 (0.10)	0.06 (0.07)	0 (0)	0 (0)	0 (0)	0 (0)	0 (0)	0.02 (0.03)
Curly	0.28 (0.42)	0.31 (0.28)	0.36 (0.19)	0.38 (0.15)	0.21 (0.15)	0.28 (0.21)	0.13 (0.11)	0.30 (0.26)	0 (0)	0.07 (0.07)	0 (0)	0.15 (0.30)
Maite	0.36 (0.32)	0.17 (0.14)	0.21 (0.12)	0.34 (0.13)	0.10 (0.05)	0.22 (0.10)	0.11 (0.03)	0.07 (0.06)	0 (0)	0.07 (0.10)	0.03 (0.04)	0.18 (0.31)
Lee	0.34 (0.17)	0.25 (0.12)	0.44 (0.03)	0.46 (0.13)	0.54 (0.21)	0.58 (0.15)	0.32 (0.28)	0.04 (0.07)	0 (0)	0 (0)	0 (0)	0.04 (0.08)
Sierra	0.19 (0.17)	0.10 (0.11)	0.05 (0.04)	0.02 (0.04)	0.02 (0.03)	0.06 (0.06)	0.02 (0.03)	0 (0)	0 (0)	0 (0)	0 (0)	0.02 (0.04)
Blunt	0.29 (0.42)	0.22 (0.31)	0.35 (0.30)	0.46 (0.07)	0.32 (0.26)	0.25 (0.01)	0.10 (0.10)	0 (0)	0 (0)	0.15 (0.10)	0.04 (0.08)	0 (0)
Marlen	0.14 (0.12)	0.22 (0.02)	0.37 (0.32)	0.28 (0.13)	0.09 (0.15)	0 (0)	0.28 (0.23)	0.03 (0.06)	0 (0)	0 (0)	0.05 (0.04)	0.14 (0.14)
Pointer	0.30 (0.32)	0.51 (0.15)	0.52 (0.21)	0.55 (0.02)	0.31 (0.11)	0.39 (0.35)	0.22 (0.20)	0.04 (0.03)	0 (0)	0 (0)	0 (0)	0.14 (0.17)

Sickle	0.17 (0.02)	0.10 (0.14)	0.22 (0.17)	0.33 (0.26)	0.19 (0.19)	0.20 (0.26)	0.27 (0.27)	0.31 (0.27)	0 (0)	0.11 (0.15)	0.10 (0.18)	0.25 (0.36)
Trailer	0.36 (0.07)	0.36 (0.14)	0.55 (0.15)	0.30 (0.03)	0.20 (0.19)	0.24 (0.22)	0.32 (0.13)	0.13 (0.10)	0 (0)	0 (0)	0 (0)	0 (0)
Twist	0.06 (0.08)	0.08 (0.11)	0.46 (0.50)	0.22 (0.28)	0.20 (0.17)	0.08 (0.09)	0.05 (0.09)	0.09 (0.11)	0 (0)	0 (0)	0 (0)	0 (0)
Moana	0.12 (0.16)	0.19 (0.27)	0.47 (0.37)	0.29 (0.14)	0.15 (0.14)	0.27 (0.42)	0.10 (0.17)	0.06 (0.05)	0 (0)	0 (0)	0 (0)	0 (0)
Tip	0.48 (0.24)	0.67 (0.08)	0.69 (0.08)	0.36 (0.14)	0.24 (0.30)	0.28 (0.26)	0.10 (0.10)	0.06 (0.05)	0 (0)	0.11 (0.15)	0 (0)	0.22 (0.38)
Brenda	0.23 (0)	0.39 (0.11)	0.41 (0.23)	0.21 (0.24)	0.13 (0.16)	0.23 (0.25)	0.25 (0.28)	0.35 (0.31)	0 (0)	0 (0)	0.05 (0.04)	0 (0)
Big Mama	0 (0)	0.03 (0.05)	0 (0)	0.06 (0.10)	0.04 (0.06)	0 (0)	0 (0)	0.02 (0.04)	0 (0)	0 (0)	0 (0)	0 (0)
Trevally	0.33 (0.03)	0.26 (0.28)	0.03 (0.04)	0.03 (0.04)	0.17 (0.20)	0.32 (0.37)	0.26 (0.01)	0.28 (0.32)	0 (0)	0 (0)	0 (0)	0 (0)
Nani	0.03 (0.04)	0.43 (0.06)	0.34 (0.06)	0.46 (0.10)	0.15 (0.22)	0.40 (0.30)	0.07 (0.08)	0.07 (0.06)	0 (0)	0.11 (0.15)	0.07 (0.07)	0.02 (0.04)
Lill	0 (0)	0 (0)	0 (0)	0 (0)	0 (0)	0.05 (0.08)	0.17 (0.25)	0.15 (0.25)	0.13 (0.18)	0.04 (0.05)	0 (0)	0 (0)
Wave	0.15 (0.21)	0.24 (0.13)	0.18 (0.25)	0.13 (0.19)	0.31 (0.32)	0.18 (0.09)	0.34 (0.09)	0.38 (0.31)	0.25 (0.35)	0.18 (0.25)	0.03 (0.04)	0.07 (0.12)
Gill	0.06 (0.08)	0.07 (0.09)	0.03 (0.04)	0.18 (0.26)	0.19 (0.27)	0 (0)	0.14 (0.19)	0.26 (0.06)	0.25 (0.35)	0 (0)	0 (0)	0 (0)
Junior	0.03 (0.04)	0.07 (0.09)	0.18 (0.25)	0.18 (0.26)	0.39 (0.43)	0.49 (0.36)	0.17 (0.11)	0.20 (0.28)	0.50 (0.71)	0 (0)	0.07 (0.12)	0 (0)
Naughtylus	0.30 (0.01)	0.28 (0.07)	0.41 (0.08)	0.18 (0.26)	0.23 (0.33)	0.38 (0.53)	0.14 (0.19)	0.16 (0.08)	0 (0)	0.36 (0)	0.22 (0.38)	0.11 (0.09)
