# Supplementary material for: Opportunistic Visitors: Long-Term Behavioural Response of Bull Sharks to Food Provisioning in Fiji
Source: PLoS One. 2013 Mar 13;8(3):e58522. doi: 10.1371/journal.pone.0058522 (PMC3596312; doi:10.1371/journal.pone.0058522)
Supplement: Table S3 — Minimum, maximum and mean (±SD) stomach temperatures (°C) and depths (m) recorded by transmitters with temperature or pressure sensors. (PDF) [file pone.0058522.s008.pdf]

Table S3. Minimum, maximum and mean (±SD) stomach temperatures (°C) and depths (m) recorded by transmitters with temperature or pressure sensors.

Transmitter ID	Minimum	Maximum	Mean
**Stomach temperature**			
46	26.22	27.48	26.65 (0.30)
47	25.91	26.85	26.54 (0.20)
50	26.54	26.69	26.64 (0.08)
69	25.44	29.36	27.84 (0.91)
70	24.81	29.05	27.07 (1.04)
71	27.32	29.05	28.58 (0.38)
72	27.01	31.24	28.38 (0.50)
76	25.6	29.05	27.81 (0.73)
77	27.95	29.2	28.52 (0.29)
81	25.28	29.67	27.63 (0.62)
82	26.69	28.58	27.80 (0.46)
84	25.6	27.95	26.95 (0.70)
**Depth**			
86	1.8	103.7	39.6 (12.7)
89	7.3	146.5	36 (14.1)
91	8.2	123.7	38.7 (15.1)
93	5.5	78.2	31.3 (8.7)
